# The Impacts of the COVID-19 Pandemic on HIV Testing Utilization Among Men Who Have Sex With Men in China: Cross-sectional Online Survey

**DOI:** 10.2196/30070

**Published:** 2022-05-25

**Authors:** Ke Chun Zhang, Yuan Fang, He Cao, Hongbiao Chen, Tian Hu, Ya Qi Chen, Xiaofeng Zhou, Zixin Wang

**Affiliations:** 1 Longhua District Center for Disease Control and Prevention Shenzhen China; 2 Department of Health and Physical Education Education University of Hong Kong Hong Kong Hong Kong; 3 JC School of Public Health and Primary Care Faculty of Medicine Chinese University of Hong Kong Hong Kong Hong Kong

**Keywords:** COVID-19, HIV testing, sexual risk behaviors, structural barriers, perception, men who have sex with men, China, MSM, HIV, testing, impact, utilization, cross-sectional, online survey, barrier, access

## Abstract

**Background:**

The COVID-19 pandemic has created disruptions in HIV prevention and sexual health services for men who have sex with men (MSM).

**Objective:**

This study compared HIV testing utilization in 3 different reference periods (period 1: before the COVID-19 outbreak, November 2019-January 2020; period 2: after the outbreak, February-April 2020; and period 3: after the pandemic was under initial control, May-July 2020). Factors associated with HIV testing utilization after the COVID-19 outbreak (combined periods 2 and 3) were also investigated.

**Methods:**

Participants were MSM aged ≥18 years living in Shenzhen, China. Those self-reporting as HIV positive were excluded. A total of 595 participants recruited through multiple sources completed a self-administered online survey during August-September 2020. HIV testing utilization after the COVID-19 outbreak was the dependent variable, and multivariate logistic regression models were fitted.

**Results:**

HIV testing utilization was significantly lower in period 2 than in period 1 (n=262 vs 363, 44.0% vs 61.0%, P<.001). However, HIV testing utilization was not significantly higher in period 3 than in period 2 (n=277 vs 262, 46.6% vs 44.0%, P=.21). The prevalence of HIV testing utilization after the COVID-19 outbreak was seen in 331 (55.6%) participants. After adjusting for significant background characteristics, condomless anal intercourse (CAI) with regular male sex partners (RPs; adjusted odds ratio [AOR] 2.15, 95% CI 1.29-3.57) and sexualized drug use (SDU; AOR 2.94, 95% CI 1.41-6.06) both before and after the COVID-19 outbreak, CAI with RPs (AOR 2.07, 95% CI 1.06-4.07) and nonregular male sex partners (NRPs; AOR 3.57, 95%CI: 1.43-8.89) only after the COVID-19 outbreak was positively associated with the dependent variable. Regarding HIV prevention service utilization, HIV testing utilization before the COVID-19 outbreak (AOR 10.75, 95% CI 7.22-16.02) and the use of sexually transmitted infection (STI) testing (AOR 7.02, 95% CI 4.10-12.02), other HIV/STI prevention (AOR 3.15, 95% CI 2.16-4.60), and preexposure prophylaxis (PrEP; AOR 3.58, 95% CI 1.54-8.34) after the COVID-19 outbreak were associated with higher HIV testing utilization. The current perceived risk of HIV infection was higher than that before the COVID-19 outbreak (AOR 1.15, 95% CI 1.01-1.30), and perceived COVID-19 preventive measures taken by HIV testing service providers to be effective (AOR 1.52, 95% CI 1.29-1.78) and perceived higher behavioral control to undergo HIV testing (AOR 1.18, 95% CI 1.00-1.40) were positively associated with HIV testing utilization. Concerns about COVID-19 infection during HIV testing (AOR 0.78, 95% CI 0.68-0.89), avoiding crowded places (AOR 0.68, 95% CI 0.48-0.98), and HIV testing service providers reducing their working hours (AOR 0.59, 95% CI 0.48-0.98) were negatively associated with the dependent variable.

**Conclusions:**

HIV testing utilization among Chinese MSM declined after the COVID-19 outbreak and did not increase after the pandemic was under initial control. Removing structural barriers to accessing HIV testing caused by COVID-19, modifying perceptions related to HIV testing, and making use of HIV self-testing (HIVST) might be useful strategies to improve HIV testing among MSM during the pandemic.

## Introduction

High coverage of HIV testing (ie, >90%) among at-risk populations is the first and a crucial step to achieve the 90-90-90 targets established by the Joint United Nations Programme on HIV/AIDS (UNAIDS), which provides a hope of ending the global HIV epidemic by 2030 [1]. International health authorities recommend men who have sex with men (MSM) to undergo HIV testing every 6 months [2,3]. In China, the HIV epidemic among MSM has been worsening over time [4]. A recent systematic review showed an overall HIV prevalence of 5.7% among MSM in China [4], whereas the HIV incidence in this group was as high as 5.6 per 100 person-years [5]. However, HIV testing coverage remained low among MSM in China (about 60% in the past year) [6].

The COVID-19 pandemic is a serious health threat worldwide, with over 147 million confirmed cases and over 3 million deaths as of April 27, 2021 [7]. The COVID-19 pandemic and its control measures (eg, lockdown, physical distancing, and closure of business) had a direct impact on HIV prevention and sexual health services for MSM. In Japan, the number of HIV tests performed by public health centers significantly declined in the second quarter of 2020 (9584 vs 35,908 in the year-before period) [8]. A similar situation was observed in Melbourne, Australia, where the number of HIV tests decreased from 16,367 in 2019 to 11,270 in 2020, a 31% reduction [9]. An online survey of a global sample of MSM showed that only 30% and 19% of participants had similar levels of access to on-site HIV testing and HIV self-testing (HIVST) during the pandemic comparing to their situation in 2019 [10]. In the United States, 18.8% of MSM had decreased access to HIV testing and 5.6% had trouble getting HIV testing after the COVID-19 outbreak [11]. There are concerns that if MSM continue to engage in sexual behaviors while having problems accessing HIV testing and other HIV or sexually transmitted infection (STI) prevention services during the pandemic, there will be a surge in new HIV cases/STIs [12]. There is a dearth of studies investigating the impact of COVID-19 on HIV testing utilization among MSM in China. To the best of our knowledge, only 1 study has looked at the difficulties in accessing HIV services in general among Chinese MSM; difficulties were reported by 56.8% of the participants [13]. The magnitude of the impact of COVID-19 on HIV testing utilization among Chinese MSM or whether service utilization will rebound after the COVID-19 pandemic is under initial control is unclear. A knowledge gap hence exists.

Understanding the barriers to HIV testing utilization during the COVID-19 pandemic is important in order to inform service planning and intervention development. Previous studies have suggested that COVID-19 control measures increase structural barriers to accessing HIV testing due to the closure of facilities providing HIV testing services, shortage of medical staff providing HIV testing, suspension of public transportation, and lockdown/travel restrictions [8,13-16]. COVID-19 also exacerbated some perceived barriers to using HIV testing, such as the fear of going to hospitals because of COVID-19, concerns about COVID-19 infection or having close contact with patients with COVID-19 during HIV testing, and perceptions that health workers were reluctant to serve them during the pandemic [8,13-16]. These factors were considered by this study.

To address these knowledge gaps, we conducted a cross-sectional online survey among MSM in China. This study had 2 objectives. The first objective was to compare self-reported utilization of any type and a specific type of HIV testing in 3 different reference periods. The first period was before the COVID-19 outbreak (November 2019-January 2020), the second was after the outbreak and before the pandemic was under initial control (February-April 2020), while the third was after the pandemic was under initial control (May-July 2020). The second objective was to investigate factors associated with self-reported utilization of any type of HIV testing after the COVID-19 outbreak (February-July 2020).

## Methods

### Study Design

We conducted a cross-sectional online survey of 595 MSM in Shenzhen, China, during August-September 2020. Shenzhen is a major metropolitan city located in Guangdong Province in southern China, with a population of 13 million in 2020.

### Participants and Data Collection

**Figure 1:**
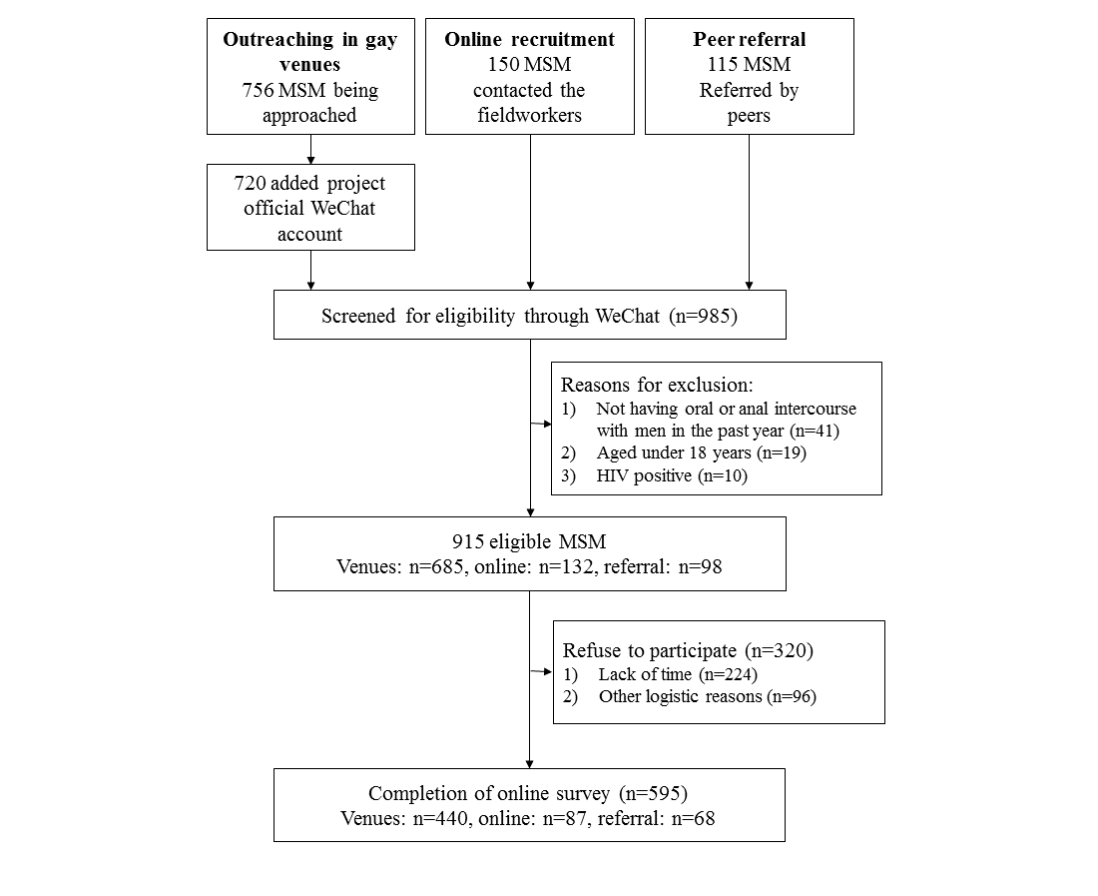
Flowchart of subject recruitment. MSM: men who have sex with men.

Participants (1) were Chinese-speaking men living in Shenzhen, (2) were aged at least 18 years, and (3) had oral or anal intercourse with at least 1 man in the past year. Those self-reporting to be HIV positive were excluded. Participants were recruited through multiple sources. Trained and experienced fieldworkers approached prospective participants in venues frequently visited by MSM (ie, bars, parks, and bathhouses) at different time slots on weekdays and weekends. The research team also conducted online outreaching by periodically posting study information on Weibo and WeChat, 2 commonly used social media platforms in China. Recruitment was supplemented by peer referrals. Fieldworkers briefed prospective participants about the study details on-site or using telephone/live chat apps and invited them to create the project’s official WeChat account. Through WeChat, fieldworkers screened the eligibility of prospective participants. Participants were assured that their identifiable information would be kept confidential, they had the right to discontinue participation in the study at any time, and their refusal or withdrawal from the study would not have any consequences. Participants signed an electronic consent form sent by WeChat. The fieldworkers approached 756 prospective participants in gay venues, 720 (95.2%) added the project official WeChat account, 685 (95.1%) were screened to be eligible through WeChat, 245 (35.8%) refused to participate, and 440 (64.2%) completed the online survey. Regarding online recruitment, 150 prospective participants contacted the fieldworkers, 132 (88%) were screened to be eligible through WeChat, 45 (34.1%) refused to participate, and 87 (65.9%) completed the survey. Of 115 prospective participants referred by peers, 98 (85.2%) were screened to be eligible through WeChat, 30 (30.6%) refused to participate and 68 (69.4%) completed the survey. A total of 595 participants completed this study. The main reasons for exclusion were (1) not having oral or anal intercourse with men in the past year (41/985, 4.2%), (2) being aged under 18 years (19/985, 1.9%), and (3) being HIV positive (10/985, 1%). The main reasons for refusals were lack of time and other logistic reasons. A flowchart of recruitment is shown in Figure 1.

We developed an online self-administered questionnaire using Questionnaire Star, a commonly used online survey platform in China. Quick response (QR) codes were generated and sent to the 595 participants through WeChat. The participants were asked to scan the QR code to complete the survey. Each mobile device was only allowed to access the online questionnaire once to avoid duplicate responses. The survey had 105 items (about 20 items per page for 5 pages), which took about 20 minutes to complete. The Questionnaire Star tool performed completeness checks before the questionnaire was submitted. The participants were able to review and change their responses through a Back button. An e-coupon of CNY 20 (US $2.97) was sent to the participants upon survey completion. All data were stored on the online server of Questionnaire Star and protected by a password. Only the corresponding author had access to the database.

### Ethical Considerations

Ethics approval was obtained from the Longhua District Centers for Disease Control and Prevention (CDC; reference: 2021009).

### Measurements

A panel consisting of 3 CDC staff, 2 public health researchers, a health psychologist, and 2 MSM volunteers was formed to develop the questionnaire used in this study. The questionnaire was pilot-tested among 10 MSM to assess clarity and readability. These 10 MSM did not participate in the actual survey. Based on their comments, the panel revised and finalized the questionnaire. The Chinese and English versions of the questionnaire are provided in Multimedia Appendix 1.

Background characteristics were collected, including age, relationship status, highest educational level attained, current employment status, monthly personal income, sexual orientation, and source of recruitment.

The dependent variable for this study was HIV testing utilization. In addition, 3 independent questions were used to assess whether participants performed a specific type of HIV testing (ie, HIV testing at community-based organizations [CBOs], public hospitals/the CDC, private hospitals, and other organizations in Shenzhen; HIV testing in a place other than Shenzhen; and home-based HIVST) in 3 reference periods. The first period was between November 2019 and January 2020. Soon after the China central government imposed a lockdown in Wuhan on January 23, 2020, Shenzhen initiated a tier 1 response (the highest level) to a major public health event on January 24, 2020 [17]. Therefore, the first reference period represented the time prior to the COVID-19 outbreak in China. The second period was between February and April 2020, after the lockdown in Wuhan was lifted in April 2020 and Shenzhen lowered its response level to tier 3 (the lowest level) in early May 2020 [17]. The second reference period hence represented the time after the COVID-19 outbreak and before the pandemic was under initial control in China. The last period was from May to July 2020, which represented the time after the COVID-19 pandemic was under initial control in China [17].

Independent variables included sexual risk behaviors, other HIV/STI prevention services, perceptions related to HIV testing, and structural barriers to HIV testing. Similar to measuring HIV testing behaviors, 3 independent questions were used to measure sexual risk behaviors and other HIV/STI prevention services in the 3 reference periods. The 4 different types of sexual risk behaviors assessed by the questionnaire were (1) condomless anal intercourse (CAI) with regular male sex partners (RPs), (2) CAI with nonregular male sex partners (NRPs), (3) CAI with male sex workers, and (4) sexualized drug use (SDU). An RP is defined as a stable boyfriend, while an NRP is defined as a man who is neither an RP nor a male sex worker. SDU is defined as the use of any of the following psychoactive substances before or during sexual intercourse: ketamine, methamphetamine, cocaine, cannabis, ecstasy, Dormicum/Halcion/Erimin 5/nonprescription hypnotic drugs, heroin, cough suppressant (not for curing cough), gamma-hydroxybutyric acid (GHB)/gamma-butyrolactone (GBL), 5-methocy-*N*,*N*-diisopropyltryptamine (Foxy), and mephedrone [18,19]. We created 4 variables comparing the presence of sexual risk behaviors before (period 1) and after (combined periods 2 and 3) the COVID-19 outbreak. The response categories of these variables were as follows: 1=no such behavior before or after the COVID-19 outbreak, 2=with such behavior only before the COVID-19 outbreak, 3=with such behavior both before and after the COVID-19 outbreak, and 4=with such behavior only after the COVID-19 outbreak.

The online survey also documented the participants’ use of other STI testing, other HIV/STI prevention services (eg, receiving free condoms, receiving peer education or education pamphlets, and attending lectures or seminars), and preexposure prophylaxis (PrEP) after the COVID-19 outbreak.

We applied the theory of planned behavior (TPB) as the theoretical framework to select perceptions related to HIV testing after the COVID-19 outbreak [20]. The TPB postulates that willingness to adopt a health-related behavior is a strong predictor of actual behavior. To form such an intention, one would evaluate the pros and cons of the behavior (positive and negative attitudes), consider whether their significant others would support such behavior (perceived subjective norm), and appraise how much control one has over the behavior (perceived behavioral control) [20]. In this study, 1 item measured the participants’ positive attitude toward HIV testing services during COVID-19 (ie, COVID-19 preventive measures taken by HIV testing service providers are effective); 2 other items measured some negative attitudes toward HIV testing services during COVID-19, such as the participants’ concerns related to the risk of contracting COVID-19 during HIV testing and inconvenience of undergoing HIV testing during the pandemic; and 2 single items measured the perceived subjective norm (ie, people who are important to you would support you to undergo HIV testing after the COVID-19 outbreak) and perceived behavioral control (ie, whether to undergo HIV testing after the COVID-19 outbreak is completely under control). The response categories for the latter 5 items were 1=strongly disagree, 2=disagree, 3=neutral, 4=agree, and 5=strongly agree. In addition, 1 item measured the perceived risk of HIV infection comparing the participants’ present situation with the situation before the COVID-19 outbreak (“When comparing your current situation versus the time before COVID-19, do you think your overall risk of HIV infection is higher, lower, or the same?”); the response categories were 1=much lower, 2=somewhat lower, 3=same, 4=somewhat higher, and 5=much higher.

The participants were also asked whether they adopted physical distancing after the COVID-19 outbreak (February-July 2020), including avoiding unnecessary travel and crowded places. Other structural barriers to utilizing HIV testing after the COVID-19 outbreak included whether HIV testing service providers were closed or had reduced their working hours and whether they had difficulty in obtaining HIVST kits and a history of home/centralized quarantine between February and July 2020.

### Statistical Analysis

HIV testing utilization was compared using McNemar tests. Since 1 of our objectives was to investigate factors associated with HIV testing utilization after the COVID-19 outbreak, we combined the utilization of any type of HIV testing in period 2 (February-April 2020) and period 3 (May-July 2020) and used it as the dependent variable in the subsequent analysis. First, associations between background characteristics and the dependent variable were analyzed using logistic regression models, and crude odds ratios (ORs) were obtained. After adjustment for those variables with *P*<0.05 in the univariate analysis, associations between independent variables of interest (HIV testing prior to the COVID-19 outbreak, other HIV/STI prevention service utilization after the COVID-19 outbreak, variables comparing the presence of sexual risk behaviors before and after the COVID-19 outbreak, perceptions related to HIV testing, and structural barriers to HIV testing after the COVID-19 outbreak) and the dependent variable were assessed by adjusted odds ratios (AORs). Each AOR was obtained by fitting a single logistic regression model, which involved 1 of the independent variables of interest and significant background variables. SPSS Statistics version 21.0 (IBM) was used for data analysis, with *P*<.05 considered statistically significant.

## Results

### Background Characteristics of the Participants

The majority of the participants were 18-30 years old (n=452, 75.9%), single (n=481, 80.8%), and employed full-time (n=433, 72.8%); had attained at least tertiary education (n=394, 66.2%), with a monthly personal income of CNY >=5000 (>=US$741.46) (n=346, 58.1%); see Table 1.

**Table 1:** Background characteristics of 595 MSM^a^ participating in a cross-sectional survey from August to September 2020.

Characteristics		Participants, n (%)
**Age (years)**		
	18-24	184 (30.9)
	25-30	268 (45.0)
	31-40	114 (19.2)
	>40	29 (4.9)
**Relationship status**		
	Single	481 (80.8)
	Cohabiting with or married to a man	92 (15.5)
	Cohabiting with or married to a woman	22 (3.7)
**Highest educational level attained**		
	Senior high school or below	201 (33.8)
	College or above	394 (66.2)
**Current employment status**		
	Full-time	433 (72.8)
	Part-time/unemployed/retired/student	162 (27.2)
**Monthly personal income^b^**		
	CNY <3000 (<US $444.87)	87 (14.6)
	CNY 3000-4999 (US $444.87-$741.31)	119 (20.0)
	CNY 5000-6999 (US $741.46-$1037.89)	118 (19.8)
	CNY 7000-9999 (US $1038.04-$1482.76)	99 (16.6)
	CNY ≥10,000 (≥US $1482.91)	129 (21.7)
	Refuse to disclose	43 (7.2)
**Sexual orientation**		
	Homosexual	427 (71.8)
	Bisexual	117 (19.7)
	Heterosexual	18 (3.0)
	Uncertain	33 (5.5)
**Source of recruitment**		
	Outreach in gay venues	440 (73.9)
	Online recruitment	87 (14.6)
	Peer referral	68 (11.4)

^a^MSM: men who have sex with men.

^b^An exchange rate of CNY 1=US $0.15 has been used.

### Frequency Distribution of Independent Variables

Relatively few participants (n=6-44, 1.0%-7.4%) reported the presence of sexual risk behaviors only after the COVID-19 outbreak (Table 2). The prevalence of sexual risk behaviors in different reference periods are shown in Multimedia Appendix 2. After the COVID-19 outbreak, 37-199 (6.2%-33.4%) participants used HIV/STI prevention services other than HIV testing (Table 3). Regarding perceptions related to HIV testing (Table 4), over half of the participants perceived their current risk of HIV infection was much/somewhat lower than that before COVID-19 (n=387, 65%) and agreed/strongly agreed that COVID-19 preventive measures taken by HIV testing service providers were effective (n=320, 53.7%). More than one-third of them had concerns related to COVID-19 infection during HIV testing (n=225, 37.9%) and the inconvenience of using HIV testing services during the pandemic (n=243, 40.8%). Regarding structural barriers to HIV testing, 58 (9.7%) and 63 (10.6%) participants reported that HIV testing service providers suspended and reduced their services, respectively, and 42 (7.1%) had difficulty in obtaining HIVST kits between February and July 2020 (Table 5).

**Table 2:** Frequency distribution of sexual risk behaviors before (November 2019-January 2020) and after (February-July 2020) the COVID-19 outbreak among 595 MSM^a^ participating in a cross-sectional survey from August to September 2020.

Independent variables		Participants, n (%)
**CAI^b^** **with RPs^c^**		
	No such behavior before or after the COVID-19 outbreak	427 (71.8)
	With such behavior only before the COVID-19 outbreak	33 (5.5)
	With such behavior both before and after the COVID-19 outbreak	91 (15.3)
	With such behavior only after the COVID-19 outbreak	44 (7.4)
**CAI with NRPs^d^**		
	No such behavior before or after the COVID-19 outbreak	509 (85.5)
	With such behavior only before the COVID-19 outbreak	19 (3.2)
	With such behavior both before and after the COVID-19 outbreak	35 (5.9)
	With such behavior only after the COVID-19 outbreak	32 (5.4)
**CAI with male sex workers**		
	No such behavior before or after the COVID-19 outbreak	576 (96.8)
	With such behavior only before the COVID-19 outbreak	5 (0.8)
	With such behavior both before and after the COVID-19 outbreak	8 (1.3)
	With such behavior only after the COVID-19 outbreak	6 (1.0)
**SDU^e^**		
	No such behavior before or after the COVID-19 outbreak	515 (86.6)
	With such behavior only before the COVID-19 outbreak	13 (2.2)
	With such behavior both before and after the COVID-19 outbreak	45 (7.6)
	With such behavior only after the COVID-19 outbreak	22 (3.7)

^a^MSM: men who have sex with men.

^b^CAI: condomless anal intercourse.

^c^RP: regular male sex partner.

^d^NRP: nonregular male sex partner.

^e^SDU: sexualized drug use.

**Table 3:** Frequency distribution of HIV/STI^a^ prevention service utilization after the COVID-19 outbreak (February-July 2020) among 595 MSM^b^ participating in a cross-sectional survey from August to September 2020.

Independent variables		Participants, n (%)
**Testing for other** **STIs**		
	No	373 (77.8)
	Yes	132 (22.2)
**Other HIV/STI prevention services (eg, receiving free condoms or peer education or education pamphlets, attending lectures or seminars)**		
	No	396 (66.6)
	Yes	199 (33.4)
**Use of PrEP^c^ before CAI^d^ with male sex workers**		
	No	558 (93.8)
	Yes	37 (6.2)

^a^STI: sexually transmitted infection.

^b^MSM: men who have sex with men.

^c^PrEP: preexposure prophylaxis.

^d^CAI: condomless anal intercourse.

**Table 4:** Frequency distribution of perceptions related to HIV testing utilization after the COVID-19 outbreak among 595 MSM^a^ participating in a cross-sectional survey from August to September 2020.

Independent variables		Participants, n (%)	Mean (SD)	
**Perceived risk of HIV infection comparing the present situation with the time before COVID-19**				2.3 (1.3)
	Much lower	218 (36.6)	N/A^b^	
	Somewhat lower	169 (28.4)	N/A	
	Same	31 (5.2)	N/A	
	Somewhat higher	143 (24.0)	N/A	
**Whether COVID-19 preventive measures taken by HIV testing services providers are effective**				3.5 (1.1)
	Strongly disagree	35 (5.9)	N/A	
	Disagree	57 (9.6)	N/A	
	Neutral	183 (30.8)	N/A	
	Agree	221 (37.1)	N/A	
	Strongly agree	99 (16.6)	N/A	
**Concern about COVID-19 infection when undergoing HIV testing**				3.1 (1.3)
	Strongly disagree	85 (14.3)	N/A	
	Disagree	90 (15.1)	N/A	
	Neutral	195 (32.8)	N/A	
	Agree	114 (19.2)	N/A	
	Strongly agree	111 (18.7)	N/A	
**Whether it is inconvenient to go to organizations providing HIV testing after the COVID-19 outbreak**				3.3 (1.2)
	Strongly disagree	59 (9.9)	N/A	
	Disagree	83 (13.9)	N/A	
	Neutral	210 (35.3)	N/A	
	Agree	124 (20.8)	N/A	
	Strongly agree	119 (20.0)	N/A	
**Whether people who are important to you support you to undergo HIV testing after the COVID-19 outbreak**				4.0 (1.0)
	Strongly disagree	28 (4.7)	N/A	
	Disagree	7 (1.2)	N/A	
	Neutral	135 (22.7)	N/A	
	Agree	212 (35.6)	N/A	
	Strongly agree	213 (35.8)	N/A	
**Whether to undergo HIV testing after the COVID-19 outbreak is completely under control**				4.0 (1.0)
	Strongly disagree	21 (3.5)	N/A	
	Disagree	14 (2.4)	N/A	
	Neutral	126 (21.2)	N/A	
	Agree	209 (35.1)	N/A	
	Strongly agree	225 (37.8)	N/A	

^a^MSM: men who have sex with men.

^b^N/A: not applicable.

**Table 5:** Frequency distribution of structural barriers among 595 MSM^a^ participating in a cross-sectional survey from August to September 2020.

Independent variables		Participants, n (%)
**Avoiding unnecessary travel**		
	No	203 (34.1)
	Yes	392 (65.9)
**Avoiding crowded places**		
	No	181 (30.4)
	Yes	414 (69.6)
**HIV testing service providers suspending their services during February-July 2020**		
	No	537 (90.3)
	Yes	58 (9.7)
**HIV testing service providers reducing their service hours during February-July 2020**		
	No	532 (89.4)
	Yes	63 (10.6)
**Difficulty in obtaining** **HIVST^b^ kits during February-July 2020**		
	No	553 (92.9)
	Yes	42 (7.1)
**History of home/centralized quarantine during February-July 2020**		
	No	504 (84.7)
	Yes	91 (15.3)

^a^MSM: men who have sex with men.

^b^HIVST: HIV self-testing.

### HIV Testing Utilization During Different Reference Periods

**Figure 2:**
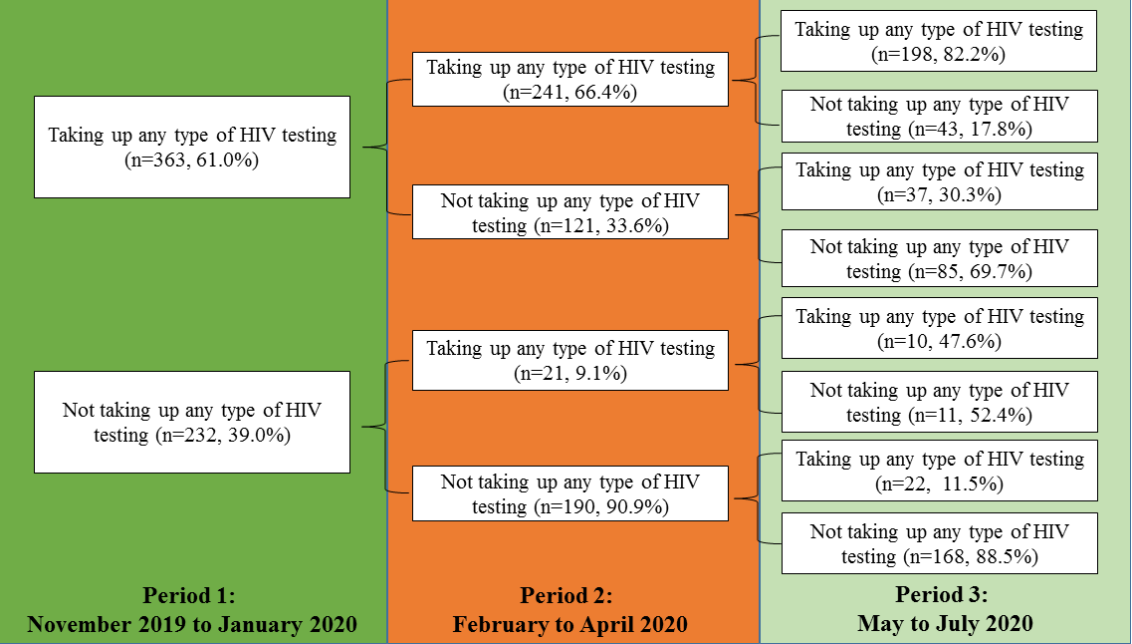
Patterns of HIV testing uptake in different reference periods.

About half of the participants underwent any types of HIV testing between February and July 2020 (n=331, 55.6%). Compared to the time before the COVID-19 outbreak (period 1, November 2019-January 2020), a significantly lower proportion of the participants underwent any type of HIV testing between February and April 2020 (period 2 vs period 1: n=262 vs 363, 44.0% vs 61.0%, *P*<.001). The proportion of testers did not increase significantly after the pandemic was under initial control in China (period 3, May-July 2020; period 2 vs period 3: n=262 vs 277, 44.0% vs 46.6%, *P*=.21; period 3 vs period 1: n=277 vs 363, 46.6% vs 61.0%, *P*<.001). We observed similar changes in the utilization of HIV testing at CBOs in Shenzhen, at public hospitals/the CDC in Shenzhen, at other organizations in Shenzhen, and in places other than Shenzhen, as well as the utilization of HIVST. In addition, 331 (55.6%) participants had undergone any type of HIV testing after the COVID-19 outbreak (combined periods 2 and 3); see Table 6. Patterns of HIV testing utilization across the study period are also shown in Figure 2.

**Table 6:** HIV testing utilization during different reference periods among 595 MSM^a^ participating in a cross-sectional survey from August to September 2020.

HIV testing locations		Participants who underwent testing, n (%)	Period 1^b^ vs period 2^c^, *P* value^d^		Period 2 vs period 3^e^, *P* value^d^		Period 3 vs period 1, *P* value^d^	
**HIV testing at CBOs^f^ in Shenzhen**				<.001		.56		<.001
	Period 1	72 (12.1)	N/A^g^		N/A		N/A	
	Period 2	41 (6.9)	N/A		N/A		N/A	
	Period 3	45 (7.6)	N/A		N/A		N/A	
	Combined periods 2 and 3	56 (9.4)	N/A		N/A		N/A	
**HIV testing at public hospitals or the CDC^h^ in Shenzhen**				<.001		.38		<.001
	Period 1	137 (23.0)	N/A		N/A		N/A	
	Period 2	77 (12.9)	N/A		N/A		N/A	
	Period 3	85 (14.3)	N/A		N/A		N/A	
	Combined periods 2 and 3	113 (19.0)	N/A		N/A		N/A	
**HIV testing at private hospitals in Shenzhen**				.08		.55		.33
	Period 1	28 (4.7)	N/A		N/A		N/A	
	Period 2	20 (3.4)	N/A		N/A		N/A	
	Period 3	23 (3.9)	N/A		N/A		N/A	
	Combined periods 2 and 3	27 (4.5)	N/A		N/A		N/A	
**HIV testing at other organizations in Shenzhen**				.002		.42		.05
	Period 1	52 (8.7)	N/A		N/A		N/A	
	Period 2	34 (5.7)	N/A		N/A		N/A	
	Period 3	39 (6.6)	N/A		N/A		N/A	
	Combined periods 2 and 3	49 (8.2)	N/A		N/A		N/A	
**HIV testing in places other than Shenzhen**				.002		.42		.05
	Period 1	129 (21.7)	N/A		N/A		N/A	
	Period 2	76 (12.8)	N/A		N/A		N/A	
	Period 3	72 (12.1)	N/A		N/A		N/A	
	Combined periods 2 and 3	98 (16.5)	N/A		N/A		N/A	
**Home-based HIVST^i^**				<.001		.83		<.001
	Period 1	260 (43.7)	N/A		N/A		N/A	
	Period 2	200 (33.6)	N/A		N/A		N/A	
	Period 3	197 (33.1)	N/A		N/A		N/A	
	Combined periods 2 and 3	241 (40.5)	N/A		N/A		N/A	
**Any type of HIV testing**				<.001		.21		<.001
	Period 1	363 (61.0)	N/A		N/A		N/A	
	Period 2	262 (44.0)	N/A		N/A		N/A	
	Period 3	277 (46.6)	N/A		N/A		N/A	
	Combined periods 2 and 3	331 (55.6)	N/A		N/A		N/A	

^a^MSM: men who have sex with men.

^b^Period 1: before the COVID-19 outbreak (November 2019-January 2020).

^c^Period 2: before COVID-19 was under initial control (February-April 2020).

^d^*P* values were obtained using McNemar tests.

^e^Period 3: after COVID-19 was under initial control (May-July 2020).

^f^CBO: community-based organization.

^g^N/A: not applicable.

^h^CDC: Centers for Disease Control and Prevention.

^i^HIVST: HIV self-testing.

### Factors Associated With HIV Testing Utilization After the COVID-19 Outbreak (February-July 2020)

In univariate analysis, participants who cohabited with or were married to a woman and identified themselves as heterosexual were less likely to undergo any type of HIV testing between February and July 2020 (Table 7).

After adjusting for these significant background characteristics, CAI with RPs and SDU both before and after the COVID-19 outbreak were associated with higher utilization of HIV testing after the COVID-19 outbreak. CAI with RPs and NRPs only after the COVID-19 outbreak was also positively associated with the dependent variable. Regarding HIV/STI prevention service utilization, utilization of HIV testing services prior to the COVID-19 outbreak was associated with higher HIV testing utilization after the COVID-19 outbreak. Users of other STI testing, other HIV/STI prevention services, and PrEP after the COVID-19 outbreak were more likely to undergo any types of HIV testing in the same period.

The current perceived risk of HIV infection was higher than that before the COVID-19 outbreak (AOR 1.15, 95% CI 1.01-1.30, *P*=.03), and perceived COVID-19 preventive measures taken by HIV testing service providers to be effective (AOR 1.52, 95% CI 1.29-1.78, *P*<.001) and perceived higher behavioral control to undergo HIV testing after the COVID-19 outbreak (AOR 1.18, 95% CI 1.00-1.40, *P*=.048) were associated with higher HIV testing utilization between February and July 2020. COVID-19 infection during HIV testing (AOR 0.78, 95% CI 0.68-0.89, *P*<.001), avoiding crowded places (AOR 0.68, 95% CI 0.48-0.98, *P*=0.04), and HIV testing service providers reducing their working hours (AOR 0.59, 95% CI 0.48-0.98, *P*=0.046) were associated with lower HIV testing utilization during the same period (Table 8).

**Table 7:** Associations between background characteristics and utilizing any HIV testing after the COVID-19 outbreak (February-July 2020) among 595 MSM^a^ participating in a cross-sectional survey from August to September 2020.

Characteristics		Prevalence of utilizing any HIV testing, n/N (%)	Crude OR^b^ (95% CI)	*P* value
**Age (years)**				
	18-24	94/184 (51.1)	1.0	N/A^c^
	25-30	157/268 (58.6)	1.35 (0.93-1.98)	.12
	31-40	60/114 (52.6)	1.06 (0.67-1.70)	.80
	>40	20/29 (69.0)	2.13 (0.92-4.92)	.08
**Relationship status**				
	Single	271/481 (56.3)	1.0	N/A
	Cohabiting with or married to a man	54/92 (58.7)	1.10 (0.70-1.73)	.68
	Cohabiting with or married to a woman	6/22 (27.3)	0.29 (0.11-0.76)	.01
**Highest educational level attained**				
	Senior high school or below	105/201 (52.2)	1.0	N/A
	College or above	226/394 (57.4)	1.23 (0.87-1.73)	.24
**Employment status**				
	Full-time	246/433 (56.8)	1.0	N/A
	Part-time/unemployed/retired/student	85/162 (52.5)	0.84 (0.58-1.21)	.34
**Monthly personal income**				
	CNY <3000 (<US $444.87)	50/87 (57.5)	1.0	N/A
	CNY 3000-4999 (US $444.87-$741.31)	60/119 (50.4)	0.75 (0.43-1.31)	.32
	CNY 5000-6999 (US $741.46-$1037.89)	64/118 (54.2)	0.88 (0.50-1.53)	.65
	CNY 7000-9999 (US $1038.04-$1482.76)	56/99 (56.6)	0.96 (0.65-1.72)	.90
	CNY ≥10,000 (≥US $1482.91)	80/129 (62.0)	1.21 (0.69-2.10)	.50
	Refuse to disclose	21/43 (48.8)	0.71 (0.34-1.47)	.35
**Sexual orientation**				
	Homosexual	241/427 (56.4)	1.0	N/A
	Bisexual	72/117 (61.5)	1.24 (0.81-1.88)	.32
	Heterosexual	3/18 (16.7)	0.15 (0.04-0.54)	.004
	Uncertain	15/33 (45.5)	0.64 (0.32-1.32)	.22
**Source of recruitment**				
	Outreach in gay venues	247/440 (56.1)	1.0	N/A
	Online recruitment	44/87 (50.6)	0.80 (0.50-1.27)	.34
	Peer referral	40/68 (58.8)	1.12 (0.67-1.87)	.68

^a^MSM: men who have sex with men.

^b^OR: odds ratio.

^c^N/A: not applicable.

**Table 8:** Factors associated with utilizing any HIV testing after the COVID-19 outbreak (February-July 2020) among 595 MSM^a^ participating in a cross-sectional survey from August to September 2020.

Factors			Crude OR^b^ (95% CI)		*P* value		AOR^c^ (95% CI)		*P* value
**CAI^d^ with RPs^e^**									
	No such behavior before or after the COVID-19 outbreak	1.0		N/A^f^		1.0		N/A	
	With such behavior only before the COVID-19 outbreak	1.71 (0.82-3.56)		.15		1.51 (0.72-3.17)		.28	
	With such behavior both before and after the COVID-19 outbreak	2.32 (1.42-3.77)		.001		2.15 (1.29-3.57)		.003	
	With such behavior only after the COVID-19 outbreak	2.09 (1.08-4.06)		.03		2.07 (1.06-4.07)		.03	
**CAI with NRPs^g^**									
	No such behavior before or after the COVID-19 outbreak	1.0		N/A		1.0		N/A	
	With such behavior only before the COVID-19 outbreak	0.63 (0.25-1.59)		.33		0.66 (0.25-1.70)		.39	
	With such behavior both before and after the COVID-19 outbreak	1.87 (0.91-3.93)		.09		1.83 (0.87-3.87)		.11	
	With such behavior only after the COVID-19 outbreak	3.75 (1.52-9.26)		.004		3.57 (1.43-8.89)		.01	
**CAI with male sex workers**									
	No such behavior before or after the COVID-19 outbreak	1.0		N/A		1.0		N/A	
	With such behavior only before the COVID-19 outbreak	3.18 (0.35-28.61)		.30		3.68 (0.40-34.19)		.25	
	With such behavior both before and after the COVID-19 outbreak	0.48 (0.11-2.01)		.31		0.46 (0.11-1.99)		.30	
	With such behavior only after the COVID-19 outbreak	0.79 (0.16-3.97)		.78		0.84 (0.16-4.34)		.84	
**SDU^h^**									
	No such behavior before or after the COVID-19 outbreak	1.0		N/A		1.0		N/A	
	With such behavior only before the COVID-19 outbreak	2.03 (0.62-6.66)		.25		1.91 (0.58-6.31)		.29	
	With such behavior both before and after the COVID-19 outbreak	3.15 (1.53-6.50)		.002		2.94 (1.41-6.06)		.004	
	With such behavior only after the COVID-19 outbreak	2.40 (0.93-6.23)		.07		2.49 (0.93-6.68)		.07	
**HIV/STI^i^ prevention service utilization**									
	Utilizing any HIV testing from November 2019 to January 2021	11.05 (7.47-16.33)		<.001		10.75 (7.22-16.02)		<.001	
	Testing for other STIs after the COVID-19 outbreak (February-July 2020)	7.18 (4.23-12.19)		<.001		7.02 (4.10-12.02)		<.001	
	Other HIV/STI prevention services (eg, receiving free condoms or peer education or education pamphlets, attending lectures or seminars) after the COVID-19 outbreak (February-July 2020)	3.14 (2.17-4.55)		<.001		3.15 (2.16-4.60)		<.001	
	Use of PrEP^j^ after the COVID-19 outbreak (February-July 2020)	3.66 (1.58-8.47)		.002		3.58 (1.54-8.34)		.002	
**Perceptions related to HIV testing** **utilization** **after the COVID-19 outbreak**									
	Perceived risk of HIV infection comparing the current situation with the time before COVID-19	1.15 (1.02-1.30)		.03		1.15 (1.01-1.30)		.03	
	COVID-19 preventive measures taken by HIV testing service providers are effective	1.55 (1.33-1.81)		<.001		1.52 (1.29-1.78)		<.001	
	Concern about COVID-19 infection when undergoing HIV testing	0.77 (0.68-0.88)		<.001		0.78 (0.68-0.89)		<.001	
	Whether it is inconvenient to go to organizations providing HIV testing after the COVID-19 outbreak	0.91 (0.80-1.04)		.16		0.88 (0.77-1.01)		.08	
	Whether people who are important to you support you to undergo HIV testing after the COVID-19 outbreak	1.05 (0.90-1.25)		.51		1.01 (0.86-1.19)		.91	
	Whether to undergo HIV testing after the COVID-19 outbreak is completely under control	1.21 (1.03-1.43)		.02		1.18 (1.00-1.40)		.048	
**Structural barriers**									
	Avoiding unnecessary travel	0.76 (0.54-1.07)		.12		0.77 (0.54-1.09)		.14	
	Avoiding crowded places	0.67 (0.47-0.95)		.02		0.68 (0.48-0.98)		.04	
	HIV testing service providers suspending their services during February-July 2020	0.58 (0.33-1.03)		.06		0.62 (0.35-1.10)		.12	
	HIV testing service providers reducing their service hours during February-July 2020	0.55 (0.31-0.96)		.04		0.59 (0.33-0.99)		.046	
	Difficulty in obtaining HIVST^k^ kits during February-July 2020	0.68 (0.35-1.30)		.24		0.69 (0.36-1.34)		.28	
	History of home/centralized quarantine during February-July 2020	0.84 (0.53-1.32)		.84		0.87 (0.55-1.38)		.55	

^a^MSM: men who have sex with men.

^b^OR: odds ratio.

^c^AOR adjusted odds ratio. The ORs were adjusted for significant background characteristics listed in Table 7 (ie, relationship status and sexual orientation).

^d^CAI: condomless anal intercourse.

^e^RP: regular male sex partner.

^f^N/A: not applicable.

^g^NRP: nonregular male sex partner.

^h^SDU: sexualized drug use.

^i^STI: sexually transmitted infection.

^j^PrEP: preexposure prophylaxis.

^k^HIVST: HIV self-testing.

## Discussion

### Principal Findings

To the best of our knowledge, this is 1 of the first studies investigating the impacts of the COVID-19 pandemic on HIV testing among MSM in China. A significant decline was observed in the utilization of facility-based HIV testing and HIVST comparing to the prepandemic era. The findings were similar to studies across countries [8-11,13]. A significant decline in sexual risk behaviors (CAI with RPs and NRPs) was also observed after the COVID-19 outbreak. Changes in sexual risk behaviors among MSM after the COVID-19 outbreak were mixed in the previous literature [21-27]. The level of sexual risk behaviors among our participants quickly rebounded to the prepandemic level after the COVID-19 pandemic was under initial control. This situation raised concerns about potential HIV/STI outbreaks among MSM in China in the postpandemic era. Currently, given the scale-up of COVID-19 vaccination, more countries are attempting to return to normal life. Our findings share some reference values for these countries regarding HIV prevention in the postpandemic era. After the control of the COVID-19 pandemic, local governments and service providers should rehire their personnel and resume their working hours for HIV prevention services. Given the implementation of physical distancing and the concerns about COVID-19 infection when using facility-based HIV testing, more efforts should be given to promote home-based HIVST with essential supporting services (eg, online counseling support and referral services for HIVST users) to mitigate the potential negative impacts caused by the pandemic.

Similar to previous findings, COVID-19 caused some structural barriers to accessing HIV testing [8,13-16]. During the pandemic, the Chinese government advocated physical distancing and recommended that people avoid unnecessary travel and crowded places [28,29]. In our study, about 70% of the participants reported avoiding crowded places after the COVID-19 outbreak. Avoiding crowded places was negatively associated with HIV testing utilization. Since facility-based HIV testing is usually provided by public hospitals, the CDC, and CBOs, it was likely that MSM would avoid these crowded places during the pandemic. About 10% of the participants reported that their HIV testing service providers reduced working hours during the pandemic, which was also a barrier. In China, public hospitals and the CDC reallocate some of the HIV prevention staff in order to implement COVID-19 prevention.

Our findings provide some empirical insights into service planning and intervention development. More attention should be given to MSM who cohabit with or are married to a woman or identify themselves as heterosexual, as in this study they reported lower HIV testing after the COVID-19 outbreak. Due to discrimination, MSM in China are sexual minorities and hidden in the population [30]. Some Chinese MSM marry a woman to conceal their homosexuality/same-sex behaviors and to deal with their parents’ expectations [30]. Since HIV is a highly stigmatized disease in China, female sexual partners knowing about the MSM’s HIV testing utilization might lead to some undesired consequences (eg, conflicts, exposure of homosexuality).

Use of HIV testing prior to the COVID-19 outbreak was associated with higher HIV testing utilization after the outbreak. Different health promotion strategies tailored to the needs of frequent and infrequent testers should be considered. Use of STI testing and other HIV/STI prevention services after the COVID-19 outbreak was also associated with higher HIV testing utilization during the same period. One explanation is that these services are usually performed simultaneously during HIV testing. COVID-19 did not have a significant impact on PrEP users, who reported higher HIV testing utilization, as they are required to undergo such tests every 3 months [31].

Maintaining or increasing sexual risk behaviors (CAI with RPs and NRPs, and SDU) after the COVID-19 outbreak was significantly associated with higher HIV testing utilization during the same period. Participants might have perceived a lower risk of HIV infection due to the decline in sexual risk behaviors after the COVID-19 outbreak and hence perceived a lower need to undergo HIV testing. The perceived higher risk of HIV infection comparing to the prepandemic era was another facilitator of HIV testing utilization. However, although their sexual risk behaviors rebounded to the prepandemic level, more than 60% of the participants perceived their risk to be lower than the prepandemic level. Facilitating MSM to have an accurate HIV risk perception may be a useful strategy. A personalized HIV risk self-assessment tool may be helpful for MSM during the pandemic, which can be adapted from the HIV risk calculator developed by Chen and Dowdy [32].

Modifying perceptions related to HIV testing after the COVID-19 outbreak may also be useful. About 40% of the participants were concerned about COVID-19 infection when undergoing HIV testing. Such concern was associated with lower HIV testing utilization. Over half of the participants perceived COVID-19 preventive measures taken by HIV testing service providers to be effective. Such perception was a facilitator of HIV testing utilization. HIV testing service providers should make their COVID-19 preventive measures transparent to potential clients to reduce their concerns. The role of HIVST became more important during the COVID-19 pandemic. Previous studies have shown that the majority of MSM were willing to utilize HIVST during the social distancing period and that they preferred home delivery of HIVST kits and support of teleconsultation [33]. Recently, a novel HIVST service was implemented among Chinese MSM. A CBO sent a free HIVST kit through mail to users and provided real-time instructions and counseling through live chat apps, making the experience of HIVST similar to facility-based HIV testing. Such a service was effective in increasing HIV testing coverage and ensuring linkage to care [34,35]. This service could also improve perceived behavioral control to undergo HIV testing after the COVID-19 outbreak, which was another facilitator. Government organizations and CBOs in China should consider allocating more resources to implement HIVST services for MSM in the postpandemic era.

### Limitations

This study had a few limitations. First, the cross-sectional study design could not adequately determine the magnitude of the impact of the COVID-19 pandemic and routine testing frequency on HIV testing utilization. However, we believe the impact of routine testing frequency would be limited. Sexually active MSM are recommended by the China CDC to undergo HIV testing every 3 months. In the presence of a window period, all people who receive a negative HIV testing result are also advised to test again 3 months afterward. In this study, the duration of each reference period was in line with the recommended interval of HIV testing for MSM.

Second, HIV testing, sexual risk behaviors, and other HIV/STI prevention service utilization in different reference periods were based on self-reported data, so recall bias existed. The participants were likely to overreport HIV testing or other HIV/STI prevention utilization and underreport sexual risk behaviors due to social desirability.

Third, participants were recruited by nonprobabilistic sampling in 1 Chinese city. Compared to other Chinese cities with a general or a lower economy, there are more organizations providing HIV testing services in Shenzhen. In addition, given the relatively high-income level of the people in Shenzhen, MSM living in the city would have lower financial barriers to using chargeable HIV testing services provided by private clinics or purchasing HIVST kits. Therefore, the findings of this study could not be applied to other Chinese cities with a general or a lower economy. The COVID-19 pandemic might have a greater impact on HIV testing services in other smaller or less developed Chinese cities.

Fourth, we were not able to obtain the characteristics of MSM who refused to participate in the study. The characteristics of those who refused to join the study might be different from the participants, so selection bias existed. The response rate was relatively high compared to online surveys on similar topics.

Fifth, the items were constructed for this study and were not validated by other studies. Moreover, we only obtained cross-sectional associations and could not establish causal relationships.

### Conclusion

In sum, utilization of facility-based and home-based HIVST among Chinese MSM declined after the COVID-19 outbreak and did not increase after the pandemic was under initial control. Removing structural barriers to accessing HIV testing, caused by COVID-19; modifying perceptions related to HIV testing; and making use of HIVST might be useful strategies to improve HIV testing among MSM during the pandemic.

## Appendix

Multimedia Appendix 1 Chinese and English versions of the survey questionnaire.

Multimedia Appendix 2 Prevalence of sexual risk behaviors and other HIV or STI prevention service utilization. STI: sexually transmitted infection.

## Abbreviations

AOR: adjusted odds ratio
CAI: condomless anal intercourse
CBO: community-based organization
CDC: Centers for Disease Control and Prevention
HIVST: HIV self-testing
MSM: men who have sex with men
NRP: nonregular male sex partner
OR: odds ratio
PrEP: preexposure prophylaxis
RP: regular male sex partner
SDU: sexualized drug use
STI: sexually transmitted infection
TPB: theory of planned behavior

## References

[1] Tsui H, Lau JTF, Xiang W, Gu J, Wang Z (2012). Should associations between HIV-related risk perceptions and behaviors or intentions be positive or negative?. PLoS One.

[2] Gu J, Lau JTF, Wang Z, Wu AMS, Tan X (2015). Perceived empathy of service providers mediates the association between perceived discrimination and behavioral intention to take up HIV antibody testing again among men who have sex with men. PLoS One.

[3] Lau JTF, Gu J, Tsui HY, Chen H, Holroyd E, Wang R, Hu X (2012). Prevalence and associated factors of condom use during commercial sex by female sex workers who were or were not injecting drug users in China. Sex Health.

[4] Dong M, Peng B, Liu Z, Ye Q, Liu H, Lu X, Zhang B, Chen J (2019). The prevalence of HIV among MSM in China: a large-scale systematic analysis. BMC Infect Dis.

[5] Zhang W, Xu J, Zou H, Zhang J, Wang N, Shang H (2016). HIV incidence and associated risk factors in men who have sex with men in Mainland China: an updated systematic review and meta-analysis. Sex Health.

[6] Zhou J, Chen J, Goldsamt L, Wang H, Zhang C, Li X (2018). HIV testing and associated factors among men who have sex with men in Changsha, China. J Assoc Nurses AIDS Care.

[7] World Health Organization Coronavirus Disease (COVID-2019) Situation Reports.

[8] Ejima K, Koizumi Y, Yamamoto N, Rosenberg M, Ludema C, Bento A, Yoneoka D, Ichikawa S, Mizushima D, Iwami S (2021). HIV testing by public health centers and municipalities and new HIV cases during the COVID-19 pandemic in Japan. J Acquir Immune Defic Syndr.

[9] Chow E, Ong J, Denham I, Fairley C (2021). HIV testing and diagnoses during the COVID-19 pandemic in Melbourne, Australia. J Acquir Immune Defic Syndr.

[10] Santos G, Ackerman B, Rao A, Wallach S, Ayala G, Lamontage E, Garner A, Holloway IW, Arreola S, Silenzio V, Strömdahl S, Yu L, Strong C, Adamson T, Yakusik A, Doan TT, Huang P, Cerasuolo D, Bishop A, Noori T, Pharris A, Aung M, Dara M, Chung SY, Hanley M, Baral S, Beyrer C, Howell S (2021). Economic, mental health, HIV prevention and HIV treatment impacts of COVID-19 and the COVID-19 response on a global sample of cisgender gay men and other men who have sex with men. AIDS Behav.

[11] Sanchez TH, Zlotorzynska M, Rai M, Baral SD (2020). Characterizing the impact of COVID-19 on men who have sex with men across the United States in April, 2020. AIDS Behav.

[12] Booton R, Fu G, MacGregor L, Li J, Ong J, Tucker J, Turner KME, Tang W, Vickerman P, Mitchell KM (2020). Estimating the impact of disruptions due to COVID-19 on HIV transmission and control among men who have sex with men in China. medRxiv.

[13] Suen YT, Chan RC, Wong EMY (2021). An exploratory study of factors associated with difficulties in accessing HIV services during the COVID-19 pandemic among Chinese gay and bisexual men in Hong Kong. Int J Infect Dis.

[14] Ponticiello M, Mwanga-Amumpaire J, Tushemereirwe P, Nuwagaba G, King R, Sundararajan R (2020). "Everything is a mess": how COVID-19 is impacting engagement with HIV testing services in rural Southwestern Uganda. AIDS Behav.

[15] Lagat H, Sharma M, Kariithi E, Otieno G, Katz D, Masyuko S, Mugambi M, Wamuti B, Weiner B, Farquhar C (2020). Impact of the COVID-19 pandemic on HIV testing and assisted partner notification services, Western Kenya. AIDS Behav.

[16] Mhango M, Chitungo I, Dzinamarira T (2020). COVID-19 lockdowns: impact on facility-based HIV testing and the case for the scaling up of home-based testing services in sub-Saharan Africa. AIDS Behav.

[17] Zou X, Wu Y, Liu X, Huang S, He J, Zhao J, Wu N, Zhang RL, Mei SJ, Liu PY, Zhang Z, Shi XL, Lyu X, Wei L, Ma QS, Lu JH, Li Y, Feng TJ, Peng CQ, Zhang SX, Xia JJ (2020). [Evaluation of the emergency response strategies and measures on the epidemic of COVID-19 in Shenzhen, China]. Zhonghua Liu Xing Bing Xue Za Zhi.

[18] Wang Z, Yang X, Mo PKH, Fang Y, Ip TKM, Lau JTF (2020). Influence of social media on sexualized drug use and chemsex among Chinese men who have sex with men: observational prospective cohort study. J Med Internet Res.

[19] Wang Z, Mo PKH, Ip M, Fang Y, Lau JTF (2020). Uptake and willingness to use PrEP among Chinese gay, bisexual and other men who have sex with men with experience of sexualized drug use in the past year. BMC Infect Dis.

[20] Ajzen I (1991). The theory of planned behavior. Organ Behav Hum Decis Process.

[21] Stephenson R, Chavanduka TMD, Rosso MT, Sullivan SP, Pitter RA, Hunter AS, Rogers E (2021). Sex in the time of COVID-19: results of an online survey of gay, bisexual and other men who have sex with men's experience of sex and HIV prevention during the US COVID-19 epidemic. AIDS Behav.

[22] Hyndman I, Nugent D, Whitlock GG, McOwan A, Girometti N (2021). COVID-19 restrictions and changing sexual behaviours in HIV-negative MSM at high risk of HIV infection in London, UK. Sex Transm Infect.

[23] de Sousa AFL, de Oliveira LB, Queiroz AAFLN, de Carvalho HEF, Schneider S, Camargo ELS, de Araújo TME, Brignol S, Mendes IAC, Fronteira I, McFarland W (2021). Casual sex among men who have sex with men (MSM) during the period of sheltering in place to prevent the spread of COVID-19. Int J Environ Res Public Health.

[24] Torres TS, Hoagland B, Bezerra DRB, Garner A, Jalil EM, Coelho LE, Benedetti M, Pimenta C, Grinsztejn B, Veloso VG (2021). Impact of COVID-19 pandemic on sexual minority populations in Brazil: an analysis of social/racial disparities in maintaining social distancing and a description of sexual behavior. AIDS Behav.

[25] Chow EPF, Hocking J, Ong J, Phillips T, Schmidt T, Buchanan A, Rodriguez E, Maddaford K, Fairley CK (2021). Brief report: changes in PrEP use, sexual practice, and use of face mask during sex among MSM during the second wave of COVID-19 in Melbourne, Australia. J Acquir Immune Defic Syndr.

[26] Gillespie D, Knapper C, Hughes D, Couzens Z, Wood F, de Bruin M, Ma R, Jones AT, Williams A, Hood K (2021). Early impact of COVID-19 social distancing measures on reported sexual behaviour of HIV pre-exposure prophylaxis users in Wales. Sex Transm Infect.

[27] Reyniers T, Rotsaert A, Thunissen E, Buffel V, Masquillier C, Van Landeghem E, Vanhamel J, Nöstlinger C, Wouters E, Laga M, Vuylsteke B (2021). Reduced sexual contacts with non-steady partners and less PrEP use among MSM in Belgium during the first weeks of the COVID-19 lockdown: results of an online survey. Sex Transm Infect.

[28] Pan Y, Fang Y, Xin M, Dong W, Zhou L, Hou Q, Li F, Sun G, Zheng Z, Yuan J, Wang Z, He Y (2020). Self-reported compliance with personal preventive measures among Chinese factory workers at the beginning of work resumption following the COVID-19 outbreak: cross-sectional survey study. J Med Internet Res.

[29] Pan Y, Xin M, Zhang C, Dong W, Fang Y, Wu W, Li M, Pang J, Zheng Z, Wang Z, Yuan J, He Y (2020). Associations of mental health and personal preventive measure compliance with exposure to COVID-19 information during work resumption following the covid-19 outbreak in China: cross-sectional survey study. J Med Internet Res.

[30] Zhu J (2017). ‘Unqueer’ kinship? Critical reflections on ‘marriage fraud’ in mainland China. Sexualities.

[31] Centers for Disease Control and Prevention (CDC) Preexposure Prophylaxis for the Prevention of HIV Infection in the United States—2017 Update. A Clinical Practice Guideline.

[32] Chen A, Dowdy DW (2014). Clinical effectiveness and cost-effectiveness of HIV pre-exposure prophylaxis in men who have sex with men: risk calculators for real-world decision-making. PLoS One.

[33] Hoagland B, Torres TS, Bezerra DR, Geraldo K, Pimenta C, Veloso VG, Grinsztejn B (2020). Telemedicine as a tool for PrEP delivery during the COVID-19 pandemic in a large HIV prevention service in Rio de Janeiro-Brazil. Braz J Infect Dis.

[34] Chan PS, Chidgey A, Lau J, Ip M, Lau JT, Wang Z (2021). Effectiveness of a novel HIV self-testing service with online real-time counseling support (HIVST-Online) in increasing HIV testing rate and repeated HIV testing among men who have sex with men in Hong Kong: results of a pilot implementation project. Int J Environ Res Public Health.

[35] Wang Z, Lau JTF, Ip M, Ho SPY, Mo PKH, Latkin C, Ma YL, Kim Y (2018). A randomized controlled trial evaluating efficacy of promoting a home-based HIV self-testing with online counseling on increasing HIV testing among men who have sex with men. AIDS Behav.

