# Supplementary material for: The Impacts of the COVID-19 Pandemic on HIV Testing Utilization Among Men Who Have Sex With Men in China: Cross-sectional Online Survey
Source: JMIR Public Health Surveill. 2022 May 25;8(5):e30070. doi: 10.2196/30070 (PMC9135116; doi:10.2196/30070)
Supplement: Multimedia Appendix 1 [file publichealth_v8i5e30070_app1.docx]

**新冠肺炎对男同生活及服务利用的影响研究**

**Impact of COVID-19 on MSM study**

Q1你现在的年龄是：_____岁

How old are you? _____years

Q2你现在的情感状况是：

What is your relationship status?

□_1_ 单身 Currently single

□_2_ 与男性同居或与男性在外国结婚 Cohabited or married with a man

□_3_ 与女性同居或已婚 Cohabited or married with a woman

Q3教育程度：

What is your education level?

□_1_ 初中或以下 Junior high or below

□_2_ 高中 Senior high

□_3_ 大专/大学或以上 College/University and above

Q4目前工作情况

What is your current employment status?

□_1_ 全职 Full-time

□_2_ 兼职 Part-time

□_3_待业/失业 Unemployed

□_4_ 退休 Retired

□_5_ 学生 Students

□_6_ 其他，请注明_____________ Others, please specific

Q5你每月平均收入在哪个范围内？

What is your monthly personal income level?

□_1_ 3000元以下 Below 3000 Chinese Yuan

□_2_ 3000至4999元 3000-4999 Chinese Yuan

□_3_ 5000至6999元 5000-6999 Chinese Yuan

□_4_ 7000至9999元 7000-9999 Chinese Yuan

□_5_ 10000元以上 10000 Chinese Yuan and above

□_6_ 无固定收入 No fixed income

□_9_ 拒绝透露 Refuse to disclose

Q6請問你的性取向是：

What is your sexual orientation?

□_1_ 同性恋 Homosexual

□_2_ 双性恋 Bisexual

□_3_异性恋 Heterosexual

□_4_ 不确定 Uncertain

Q7请问你在以下几个时间段内是否做过以下的艾滋病检测？

Did you perform the following types of HIV testing in these periods?

|  | 2019年11月  至2020年1月  November 2019 to January 2020 | 2020年2月至4月  February to April 2020 | 2020年5月至7月  May to July 2020 |
| --- | --- | --- | --- |
| A在深圳的社区组织做检测  HIV testing at community-based organization in Shenzhen | □_1_ 有 □_0_没有  □_1_ Yes □_0_ No | □_1_ 有 □_0_没有  □_1_ Yes □_0_ No | □_1_ 有 □_0_没有  □_1_ Yes □_0_ No |
| B在深圳的政府医院或者疾控做检测  HIV testing at public hospitals or Centers for Disease Control and Prevention (CDC) in Shenzhen | □_1_ 有 □_0_没有  □_1_ Yes □_0_ No | □_1_ 有 □_0_没有  □_1_ Yes □_0_ No | □_1_ 有 □_0_没有  □_1_ Yes □_0_ No |
| C在深圳私立医院做检测  HIV testing at private hospitals in Shenzhen | □_1_ 有 □_0_没有  □_1_ Yes □_0_ No | □_1_ 有 □_0_没有  □_1_ Yes □_0_ No | □_1_ 有 □_0_没有  □_1_ Yes □_0_ No |
| D在深圳的其他类型的机构做检测  HIV testing at other organizations in Shenzhen | □_1_ 有 □_0_没有  □_1_ Yes □_0_ No | □_1_ 有 □_0_没有  □_1_ Yes □_0_ No | □_1_ 有 □_0_没有  □_1_ Yes □_0_ No |
| E在深圳以外的地方做检测  HIV testing in place other than Shenzhen | □_1_ 有 □_0_没有  □_1_ Yes □_0_ No | □_1_ 有 □_0_没有  □_1_ Yes □_0_ No | □_1_ 有 □_0_没有  □_1_ Yes □_0_ No |
| F艾滋病自检（独自完成测试的整个过程，包括采集标本，进行测试和判断结果）  Home-based HIV self-testing | □_1_ 有 □_0_没有  □_1_ Yes □_0_ No | □_1_ 有 □_0_没有  □_1_ Yes □_0_ No | □_1_ 有 □_0_没有  □_1_ Yes □_0_ No |

Q8请问你最近一次艾滋病测试的结果是： □_1_ 阴性 □_2_ 阳性 □_3_ 拒绝透露

What is the results of your most recent episode of HIV testing? □_1_ Negative □_2_ Positive □_3_ Refuse to disclose

Q9请问你在以下几个时间段内是否发生过以下的行为

Did you have the following behaviors in these periods?

|  | 2019年11月  至2020年1月  November 2019 to January 2020 | 2020年2月至4月  February to April 2020 | 2020年5月至7月  May to July 2020 |
| --- | --- | --- | --- |
| A与男性固定性伴发生不用安全套的肛交  Condomless anal intercourse with regular male sex partners | □_1_ 有 □_0_没有  □_1_ Yes □_0_ No | □_1_ 有 □_0_没有  □_1_ Yes □_0_ No | □_1_ 有 □_0_没有  □_1_ Yes □_0_ No |
| B与男性非固定性伴发生不用安全套的肛交  Condomless anal intercourse with non-regular male sex partners | □_1_ 有 □_0_没有  □_1_ Yes □_0_ No | □_1_ 有 □_0_没有  □_1_ Yes □_0_ No | □_1_ 有 □_0_没有  □_1_ Yes □_0_ No |
| C与男性性工作者发生不用安全套的肛交  Condomless anal intercourse with male sex workers | □_1_ 有 □_0_没有  □_1_ Yes □_0_ No | □_1_ 有 □_0_没有  □_1_ Yes □_0_ No | □_1_ 有 □_0_没有  □_1_ Yes □_0_ No |
| D在肛交前或者过程中服用药物（例如Rush、K粉、冰、可卡因、摇头丸、海洛因、G水、零号/一号胶囊）  Use of psychoactive substances (ketamine, methamphetamine, cocaine, cannabis, ecstasy, Dormicum/Halcion/Erimin 5/non-prescription hypnotic drugs, heroin, cough suppressant (not for curing cough), gamma-hydroxybutyric acid (GHB)/gamma-butyrolactone (GBL), 5-methocy-N, N-diisopropyltryptamine (Foxy), or mephedrone) before or during anal intercourse | □_1_ 有 □_0_没有  □_1_ Yes □_0_ No | □_1_ 有 □_0_没有  □_1_ Yes □_0_ No | □_1_ 有 □_0_没有  □_1_ Yes □_0_ No |
| E做性病检测  Test for other sexually transmitted infections | □_1_ 有 □_0_没有  □_1_ Yes □_0_ No | □_1_ 有 □_0_没有  □_1_ Yes □_0_ No | □_1_ 有 □_0_没有  □_1_ Yes □_0_ No |
| F接受过其他艾滋病预防的服务，例如安全套发放，同伴教育，参加艾滋病宣传的传单和讲座  Use of other HIV/STI prevention services (e.g., receiving free condoms, peer education or education pamphlet, and attending lectures or seminars) | □_1_ 有 □_0_没有  □_1_ Yes □_0_ No | □_1_ 有 □_0_没有  □_1_ Yes □_0_ No | □_1_ 有 □_0_没有  □_1_ Yes □_0_ No |
| G服用暴露前预防用药  Use of pre-exposure prophylaxis | □_1_ 有 □_0_没有  □_1_ Yes □_0_ No | □_1_ 有 □_0_没有  □_1_ Yes □_0_ No | □_1_ 有 □_0_没有  □_1_ Yes □_0_ No |

Q10将现在的情况与疫情之前比较，总体而言你感染艾滋病的风险有什么变化？

When comparing your current situation versus the time before COVID-19, do you think your overall risk of HIV infection is higher, lower, or the same?

□_1_ 低了很多 Much lower

□_2_低了一些 Somewhat lower

□_3_ 没有变化 The same

□_4_高了一些 Somewhat higher

□_5_ 高了很多 Much higher

| Q11请问你是否同意以下关于在疫情期间做艾滋病  测试的说法  Do you agree with the following statements related to  HIV testing during the COVID-19 pandemic | **完全**  **不同意**  **Strongly disagree** | **不同意**  **Disagree** | **中立**  **Neutral** | **同意**  **Agree** | **完全**  **同意**  **Strongly agree** |
| --- | --- | --- | --- | --- | --- |
| A 你担心去做艾滋病测试的时候被传上新冠肺炎  You concern about COVID-19 infection when taking up HIV testing | □_1_ | □_2_ | □_3_ | □_4_ | □_5_ |
| B在疫情爆发后去提供测试的机构，你觉得不方便  It is inconvenient to go to organizations providing HIV testing after the COVID-19 outbreak | □_1_ | □_2_ | □_3_ | □_4_ | □_5_ |
| C对你重要的人会支持你在疫情爆发后去做艾滋病测试  People who are important to you support you to take up HIV testing after the COVID-19 outbreak | □_1_ | □_2_ | □_3_ | □_4_ | □_5_ |
| D在疫情爆发后，是否去做艾滋病测试完全在你的控制中  Whether to take up HIV testing after the COVID-19 outbreak is completely under your control | □_1_ | □_2_ | □_3_ | □_4_ | □_5_ |
| E提供艾滋病测试的机构采取的防疫措施对于预防新冠肺炎是有效的  COVID-19 preventive measures taken up by HIV testing service providers are effective | □_1_ | □_2_ | □_3_ | □_4_ | □_5_ |

| Q12在疫情期间，你有没有以下的经历？  Do you have the following experiences after the COVID-19 outbreak? | 有  Yes | 没有  No |
| --- | --- | --- |
| A提供艾滋病测试的机构停止营业  HIV testing service providers suspended their services | □_1_ | □_2_ |
| B提供艾滋病测试的机构缩短了服务时间  HIV testing service providers reduced their service hours | □_1_ | □_2_ |
| C无法获得艾滋病自检试剂  Difficult to obtain HIV self-testing kits | □_1_ | □_2_ |
| G自己被居家隔离/强制隔离  History of home or centralized quarantine | □_1_ | □_2_ |
| H尽可能在留家中避免不必要的外出  Avoiding unnecessary travel | □_1_ | □_2_ |
| I避免去人多的地方  Avoiding crowed places | □_1_ | □_2_ |

**问卷结束**

**End of the questionnaire**
