# Supplementary material for: The Impacts of the COVID-19 Pandemic on HIV Testing Utilization Among Men Who Have Sex With Men in China: Cross-sectional Online Survey
Source: JMIR Public Health Surveill. 2022 May 25;8(5):e30070. doi: 10.2196/30070 (PMC9135116; doi:10.2196/30070)
Supplement: Multimedia Appendix 2 [file publichealth_v8i5e30070_app2.docx]

Multimedia appendix 2. Prevalence of sexual risk behaviors and other HIV or STI prevention service utilization in different reference periods

|  | n (%) Yes | Period 1  versus  Period 2 | Period 2  versus  Period 3 | Period 3 versus  Period 1 |
| --- | --- | --- | --- | --- |
|  |  | *p* value ^a^ | *p* value ^a^ | *p* value ^a^ |
| **Occurrence of different sexual risk behaviors** |  |  |  |  |
| Condomless anal intercourse with regular male sex partners |  | 0.001 | 0.18 | 0.09 |
| Period 1 ^b^ | 124 (20.8) |  |  |  |
| Period 2 ^c^ | 96 (16.1) |  |  |  |
| Period 3 ^d^ | 108 (18.2) |  |  |  |
| Combine Period 2 and 3 | 135 (22.7) |  |  |  |
| Condomless anal intercourse with non-regular male sex partners |  | 0.03 | 0.03 | 1.00 |
| Period 1 ^b^ | 54 (9.1) |  |  |  |
| Period 2 ^c^ | 38 (6.4) |  |  |  |
| Period 3 ^d^ | 53 (8.9) |  |  |  |
| Combine Period 2 and 3 | 67 (11.3) |  |  |  |
| Condomless anal intercourse with male sex workers |  | 0.58 | 1.00 | 0.34 |
| Period 1 ^b^ | 13 (2.2) |  |  |  |
| Period 2 ^c^ | 10 (1.7) |  |  |  |
| Period 3 ^d^ | 9 (1.5) |  |  |  |
| Combine Period 2 and 3 | 14 (2.4) |  |  |  |
| Sexualized drug use |  | 0.09 | 0.06 | 1.00 |
| Period 1 ^b^ | 57 (9.6) |  |  |  |
| Period 2 ^c^ | 47 (7.9) |  |  |  |
| Period 3 ^d^ | 58 (9.7) |  |  |  |
| Combine Period 2 and 3 | 67 (11.3) |  |  |  |
|  |  |  |  |  |
| **Utilization of other HIV/STI prevention services** |  |  |  |  |
| Testing for other sexually transmitted infections |  | <0.001 | 0.35 | 0.002 |
| Period 1 ^b^ | 131 (22.0) |  |  |  |
| Period 2 ^c^ | 91 (15.3) |  |  |  |
| Period 3 ^d^ | 100 (16.8) |  |  |  |
| Combine Period 2 and 3 | 132 (22.2) |  |  |  |
| Other HIV/STI prevention services (e.g., receiving free condoms or peer education or education pamphlets, attending lectures or seminars) |  | <0.001 | 0.72 | <0.001 |
| Period 1 ^b^ | 243 (40.8) |  |  |  |
| Period 2 ^c^ | 162 (27.2) |  |  |  |
| Period 3 ^d^ | 166 (27.9) |  |  |  |
| Combine Period 2 and 3 | 199 (33.4) |  |  |  |
| Use of pre-exposure prophylaxis |  | 0.09 | 0.45 | 0.65 |
| Period 1 ^b^ | 34 (5.7) |  |  |  |
| Period 2 ^c^ | 27 (4.5) |  |  |  |
| Period 3 ^d^ | 31 (5.2) |  |  |  |
| Combine Period 2 and 3 | 37 (6.2) |  |  |  |

^a^ P values were obtained by using McNemar tests

^b^ Period 1: before the COVID-19 outbreak (November 2019 to January 2020)

^c^ Period 2: before the COVID-19 was under initial control (February to April 2020)

^d^ Period 3: after the COVID-19 was under initial control (May to July 2020)
